# Exercise training improves vascular function in adolescents with type 2 diabetes

**DOI:** 10.14814/phy2.12713

**Published:** 2016-02-17

**Authors:** Louise H. Naylor, Elizabeth A. Davis, Rachelle J. Kalic, Niru Paramalingam, Mary B. Abraham, Timothy W. Jones, Daniel J. Green

**Affiliations:** ^1^ School of Sport Science, Exercise & HealthThe University of Western Australia, CrawleyAustralia; ^2^ School of Paediatric and Child HealthThe University of Western Australia, CrawleyAustralia; ^3^ Telethon Kids InstituteThe University of Western Australia, SubiacoAustralia; ^4^ Department of Endocrinology and DiabetesPrincess Margaret Hospital, SubiacoAustralia; ^5^ Research Institute for Sport and Exercise SciencesLiverpool John Moores UniversityLiverpool, United Kingdom

**Keywords:** Diabetes, exercise training, vascular endothelium

## Abstract

The impact of exercise training on vascular health in adolescents with type 2 diabetes has not been previously studied. We hypothesized that exercise training would improve micro‐ and macrovascular health in adolescents with type 2 diabetes. Thirteen adolescents (13–21 years, 10F) with type 2 diabetes were recruited from Princess Margaret Hospital. Participants were randomized to receive either an exercise program along with standard clinical care (*n* = 8) or standard care alone (*n* = 5). Those in the intervention group received 12 weeks of gym‐based, personalized, and supervised exercise training. Those in the control group were instructed to maintain usual activity levels. Assessments were conducted at baseline and following week 12. The exercise group was also studied 12 weeks following the conclusion of their program. Assessments consisted of conduit artery endothelial function (flow‐mediated dilation, FMD) and microvascular function (cutaneous laser Doppler). Secondary outcomes included body composition (dual‐energy X‐ray absorptiometry, DXA), glycemic control (whole body insulin sensitivity, M) assessed using the euglycemic–hyperinsulinemic clamp protocol, cardiorespiratory fitness ($\dot{V}O_{2\mathrm{peak}}$), and muscular strength (1RM). Exercise training increased FMD (*P *<* *0.05), microvascular function (*P *<* *0.05), total lean mass (*P *<* *0.05), and muscle strength (*P < *0.001). There were no changes in cardiorespiratory fitness, body weight, BMI, or M. In the control group, body weight (*P *<* *0.01), BMI (*P *<* *0.01), and total fat mass (*P *<* *0.05) increased. At week 24, improvements in vascular function were reversed. This study indicates that exercise training can improve both conduit and microvascular endothelial function and health, independent of changes in insulin sensitivity in adolescents with type 2 diabetes.

## Introduction

Type 2 diabetes in the young is an emerging pediatric health concern with profound societal implications (Riddoch 1998). The prevalence of type 2 diabetes in adolescents increased approximately 10‐fold between 1996 and 2002 (Hotu et al. 2004), with a report from our group indicating a similar trend (Davis et al. 2002). Insulin resistance, formerly rarely diagnosed prior to adulthood, is now estimated to be present in 20–25% of obese children and adolescents (Sinha et al. 2002), with type 2 diabetes prevalent in >5% of this group.

Of particular concern in this high‐risk cohort is the rapid appearance and progression of vascular disease. We have previously published data indicating that early signs of vascular dysfunction are apparent in children and adolescents with obesity and type 2 diabetes, compared to both lean and BMI‐matched counterparts (Naylor et al. 2011). Adolescent onset type 2 diabetes is often aggressive, with earlier onset of complications than adult‐onset type 2 diabetes or childhood type 1 diabetes. For example, in Japanese children with type 2 diabetes, incipient retinopathy was detected in 36% of the cases at the time of diagnosis and in 39% of the cases at 2 years follow‐up, while microalbuminuria was observed in 39% at 2 years (Yokoyama et al. 2000). Similarly, 22% of Pima Indian children with type 2 diabetes had microalbuminuria, and at follow‐up (20–29 years) 60% had developed microalbuminuria and 17% macroalbuminuria (Krakoff et al. 2003). Similarly, data from Australia indicate that young people with type 2 diabetes had significantly higher rates of microalbuminuria and hypertension, despite a shorter duration of diabetes and lower HbA1c than those with type 1 diabetes (Eppens et al. 2006). Indeed, an International Diabetes Federation consensus statement concluded that the “onset of diabetes in childhood or adolescence heralds many years of disease and an increased risk that the full range of both micro‐ and macrovascular complications will occur when affected individuals are still relatively young” (Alberti et al. 2004). However, little is known about the most effective treatment approaches to reduce cardiovascular disease in these young patients (Kaufman 2002; Copeland et al. 2013), in whom surgical and medication strategies are not without risk and largely unproven in terms of long‐term efficacy.

Exercise is a cornerstone of diabetes prevention and management in adults (Colberg et al., 2010; American Diabetes Association, 2011) and has been demonstrated to improve endothelial function in both resistance (forearm plethysmography and infusions of acetylcholine, ACh) and conduit vessels (flow‐mediated dilation, FMD) in adults with type 2 diabetes (Maiorana et al. 2001). Similarly, Colberg et al. (2002) assessed skin blood flow responses before and after an exercise intervention in adults with type 2 diabetes and reported that exercise training enhanced cutaneous microvascular function, assessed using laser Doppler. Such studies have not, however, been performed in children or adolescents and this is highly relevant since interventions that modify vascular function and health in the early, preclinical stages of atherosclerotic disease (Ross 1993) hold the potential to contribute to prolonged retardation of lesion development and clinical progression. From the experimental viewpoint, type 2 diabetes in children also offers a model to improve our understanding of primary prevention, independent of the influence of aging and comorbidities that are common in adults.

To date, no studies have assessed whether exercise interventions can induce similar improvements in function as those seen in adults. Of the few studies carried out in adolescents, mixed results have been reported following intensive lifestyle modifications (Reinehr et al. 2008; TODAY study group, 2012; Copeland et al. 2013). In addition, few studies have investigated the effects of exercise training on aspects of the insulin resistance syndrome in obese children (Ferguson et al. 1999; Bell et al. 2007) and studies are altogether lacking in type 2 diabetic youth. We hypothesized that exercise training would enhance conduit and microvessel endothelial function, glycemic control, body composition, and cardiorespiratory fitness in adolescents with type 2 diabetes compared to those who only received standard care.

## Materials and Methods

### Ethical approval

All study procedures were approved by the Human Research Ethics Committee of Princess Margaret Hospital for Children and the University of Western Australia and the study conformed to the Declaration of Helsinki. All participants provided written consent and, for those participants <18 years, their parents also gave written informed consent.

### Subject characteristics

Thirteen participants (13–21 years) diagnosed with type 2 diabetes, who were free from preexisting type 1 diabetes or diagnosed cardiovascular disease, were recruited to this study from the Department of Endocrinology and Diabetes at Princess Margaret Hospital for Children. The diagnosis of type 2 diabetes was made on clinical and biochemical grounds, according to ISPAD guidelines (Zeitler et al. 2014).

### Study design

All measures were collected prior to and following a 12‐week intervention period. After baseline assessment, block randomization was used to allocate participants to receive an exercise intervention along with standard clinical care or to act as controls who received standard clinical care alone (education about lifestyle changes with or without metformin and/or insulin). Additionally, to determine whether changes induced by the training program were maintained, measures were repeated in the exercise group following a further 12 week period after the formal training program ceased. The use of block randomization ensured exercise in small groups (maximum four per group), allowing for direct supervision and individualization of programs to all participants.

### Exercise intervention

Participants attended 3, 1‐h, exercise training sessions per week for a period of 12 weeks. An experienced Advanced Exercise Physiologist (AEP) closely supervised all exercise sessions to ensure compliance with the prescribed exercise programs. All sessions used a combined aerobic (65–85% of HR_max_) and resistance (55–70% MVC) training regimen, with HR_max_ determined from the $\dot{V}O_{2\max}$ assessment, and MVC via strength assessments (see below). Training volume was gradually and progressively increased. Regular stretching and core strengthening exercises were included in the sessions to minimize the risk of injury.

### Experimental measures

Experimental measures included the assessment of conduit artery endothelial function using the flow‐mediated dilation (FMD) technique and microvascular assessment via cutaneous laser Doppler using combined pharmacological and heating interventions. Glycemic control was assessed via the gold standard hyperinsulinemic–euglycemic clamp (clamp), while body composition was assessed using dual‐energy X‐ray absorptiometry (DXA). Cardiorespiratory fitness ($\dot{V}O_{2\mathrm{peak}}$) was evaluated using a graded exercise test and muscular strength using one‐repetition maximum (1RM) tests. All assessments were conducted in a quiet, temperature controlled environment and all studies were conducted at the same time of the day to eliminate possible circadian variations (Jones et al. 2012). All postintervention assessments were performed at least 72 h postexercise to ensure that acute exercise did not impact upon the results.

### Assessment of conduit artery endothelial function: flow‐mediated dilation (FMD)

Participants arrived at the cardiovascular laboratory following at least 6 h of fasting, having abstained from alcohol and/or caffeine and exercise for 72 h prior to testing. After a 20‐min rest period, the brachial artery diameter response to FMD was assessed, using a 12L5v linear array transducer, attached to a high‐resolution ultrasound machine (t3200; Terason, Burlington, MA). Detailed descriptions of this technique are provided elsewhere (Woodman et al. 2001; Thijssen et al. 2011). Briefly, participants lay supine with their arms extended at ~80° from their torso. A rapid inflation/deflation pneumatic cuff (AG 101 Hokanson, Bellevue) was placed around the arm immediately distal to the olecranon process. When an optimal B‐mode image was obtained, images were collected using an insonation angle (always <60°), which did not vary during each study or within individuals across the intervention. Baseline images were recorded for 1 min, before the forearm cuff was inflated to 220 mmHg for 5 min. Recording resumed 30 sec prior to cuff deflation and continued for 5 min postdeflation. Flow‐mediated dilation is presented as the relative (%) rise from the preceding baseline diameter.

### Assessment of microvascular function: microdialysis and laser Doppler flowmetry

The microdialysis technique involving prolonged nonpainful local heating, as described by Black et al. (2008) was adopted for this study and performed during euglycemic–hyperinsulinemic clamps (see below for further details) to ensure that potential variance in glycemic control did not influence the results. After lying on the bed comfortably with the two catheters inserted on the right arm for the glycemic clamps, two very fine microdialysis fibers (Linear 30, CMA Microdialysis Ltd., Stockholm, Sweden), containing 10‐mm long 6‐kDa membranes, were placed in the dermal layer of the skin following initial placement of a 21‐gauge needle. The needles were then removed and the embedded fibers perfused with Ringer's (site 1) or the nitric oxide (NO) blocker *N* ^G^‐monomethyl‐l‐arginine (LNMMA, 10 mmol; site 2) at a rate of 5 *μ*L min^−1^ using a microinfusion pump (Model 11 plus, Harvard Apparatus, MA). LNMMA induces vasoconstriction of the microcirculation by blocking NO synthase and production. Skin perfusion was measured over both microdialysis sites using a Perimed System 5000 with integrated laser Doppler probes each consisting of a 7 laser array (Model 413, Periflux 5001 System, Perimed AB, Sweden), above each microdialysis fiber. The laser Doppler probe signals were continuously monitored via a software chart recorder (LabChart 7). At each designated study time point (5 min intervals), skin blood flow was assessed by averaging laser Doppler flux (LDF), measured in perfusion units (PU), over a stable 2‐min period. These data were subsequently converted to cutaneous vascular conductance (CVC), calculated as PU ÷ MAP (mmHg), where MAP (mean arterial pressure) was derived from contemporaneous automated blood pressure (Critikon DINAMAP Vital Signs Monitor 8100) measures.

**Figure 1:**
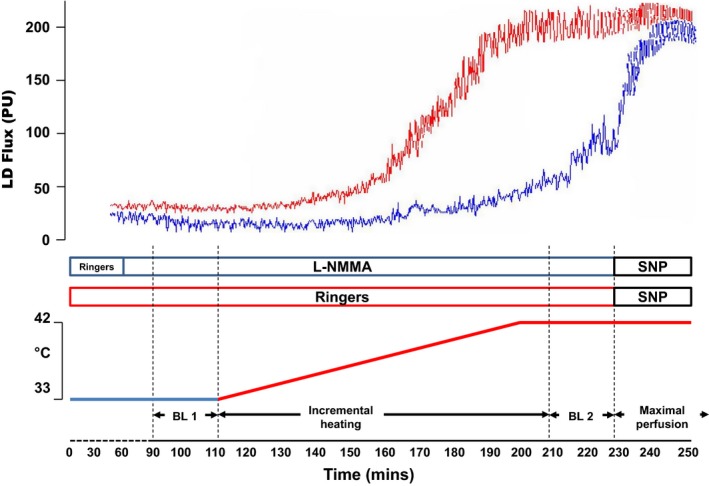
Schematic of the methodology employed to assess skin blood flow. Two sites were assessed, one with continuous infusion of Ringers, and one site with the NO‐blocker, L‐NMMA. Following a 90min stabilisation period, both sites were clamped at 33 degrees for a period of 20min (baseline 1, BL 1). Participants were then subjected to the prolonged localised heating protocol where temperature was increased from 33‐42 degrees at a rate of 0.5 degrees every 5min (90min), as described by Black et al. (2008). Once 42 degrees was achieved, both sites were held at this temperature (baseline 2, BL 2). Finally, SNP was administered to both sites to determine maximal blood flow response.

Following the equilibration period involving Ringer's or LNMMA infusions, skin blood flow at 33°C was recorded for a 20‐min period. Both probes were then heated gradually, using local heating disks surrounding the Doppler probes and overlaying the microdialysis sites, from 33°C to 42°C at a rate of 0.5°C every 5 min to a temperature of 42°C (90 min), as described by Black et al. (2008). Both sites were continuously heated at 42° for the remainder of the study. At the end of the study, sodium nitroprusside (56 mmol L^−1^) was infused through both sites to stimulate a maximal skin blood flow response (Cracowski et al. 2006). Figure 1 shows details of the study design pertaining to assessment of NO‐mediated microvascular function. By comparing the magnitude of CVC% increase with heating in the presence of LNMMA at site 2 to the magnitude of increase with heating in the Ringers’ solution (site 1), we were able to identify the magnitude of the NO contribution to the local heating response.

### Assessment of insulin sensitivity

Participants attended the research unit for the clamp study, having fasted from 10 pm of the previous night. They were advised to omit metformin in the 24 h preceding the study and insulin on the morning of the study. They were also advised to avoid exercise for 72 h prior to testing to account for the effect of acute exercise on glycemic control.

Two intravenous catheters (BD Insyte I.V. Catheter, 22GA 1.00IN; 0.9 × 25 mm) were inserted into superficial veins of the right arm. The first catheter, in the antecubital fossa, was used for infusion of insulin and glucose, while the second catheter, in a superficial digital vein, was used to measure blood glucose to guide the rate of glucose infusion. Following insertion of catheters, target plasma glucose levels at each stage of the clamp were achieved by titrating the rate of infusion of a 20% dextrose solution while maintaining constant rate of insulin (A 1:1 dilution of 60U Humalog in 60 mL of 0.9% saline solution) at 60 mU m^−2^ min^−1^. Blood samples were taken every 5–10 min and analyzed at the bedside using a YSI2300 glucose analyzer (YSI2300. Yellow Springs Instrument, OH). Blood glucose concentrations were stabilized at 5.5 (±0.1) mmol L^−1^ over a period of 90 min. Following this stabilization period, all participants were clamped at euglycemia (~5.5 mmol L^−1^) using the hyperinsulinemic euglycemic clamp technique first described by DeFronzo et al. (1979) for 60 min for the assessment of insulin sensitivity.

### Assessment of body composition

On arrival to the laboratory, height and weight were measured and BMI and BMI *z*‐scores calculated. Whole body dual emission X‐ray absorptiometry (DXA) assessment (Lunar Prodigy, GE Medical Systems, Madison, WI) was used to determine body composition. Specifically, total fat mass, total lean body mass, and body fat percentage were assessed. Regional components of body composition were determined offline using dedicated software.

### Assessment of aerobic fitness

Graded exercise tests were performed on a bicycle ergometer (Monarck). The test consisted of 3‐min exercise epochs, increasing until volitional exhaustion. Expired air was analyzed for O_2_ and CO_2_ concentrations (Ametek Gas Analyzers, Applied Electrochemistry, SOC S‐3A/1 and COV CD‐3A, Pittsburgh, PA) and ventilation (VE) was recorded at 15 sec intervals using a turbine ventilometer (Morgan, 225A, Kent, England). Prior to and following each test, the ventilometer and gas analyzers were calibrated according to the manufacturer's instructions using a 1L syringe and gases of known concentration (BOC Gases, Chatswood, Australia). Peak oxygen consumption ($\dot{V}O_{2\mathrm{peak}}$) was determined by summing the four highest consecutive 15 sec $\dot{V}O_{2}$ values in each workload. Heart rate was continually monitored using a Polar Heart Rate Monitor (Polar F1, Finland) throughout the test and recorded in the last 10 sec of each workload.

### Assessment of muscular strength

Maximal upper and lower body strength was determined using upper body exercises (bench press, pectoral strength, bicep curl) and squat exercises according to a 1RM protocol. Participants were briefed on correct form for each exercise and performed familiarization lifts. A warm‐up of 10 repetitions at 50% of their predicted 1RM was given followed by five repetitions at 70%, three repetitions at 80%, and one repetition at 90% of predicted 1RM. Participants were then given three attempts to determine their actual 1RM. A recovery of 5 min was given between efforts.

### Statistics

Statistical analyses were performed using SPSS 17.0 (SPSS, Chicago, IL). All data are reported as mean ± SE, unless stated otherwise. Statistical significance was assumed at *P *<* *0.05. Two‐way ANOVA was used to determine the effect of exercise training on all outcome measures. Post hoc *t* tests were conducted to further interrogate statistically significant ANOVA results. One‐way ANOVA was used to determine whether the impact of training persisted beyond the formal training period.

## Results

There were no differences in terms of subject characteristics between the groups at baseline, indicating that the groups were similar at entry (Table 1). There were no differences between the exercise and control groups in resting HR (79 ± 5 vs. 71 ± 3 bpm, *P* = 0.26), resting SBP (128 ± 4 vs. 121 ± 4 mmHg, *P* = 0.18), resting DBP (86 ± 3 vs. 81 ± 4 mmHg, *P* = 0.30), or HbA1c (8.8 ± 1.0 vs. 6.6 ± 0.8%, *P* = 0.11). Four participants in the exercise group and three in the control group were prescribed metformin, while two participants in the exercise group were using a combination of metformin and insulin. The remaining participants were not prescribed any medication for their diabetes. Three participants, two from the exercise, and one from the control group, opted not to participate in the glycemic clamp or microvascular assessments.

**Table 1:** Baseline and week 12 data

	Exercise group		Control group		*P* values
	Pre	Post	Pre	Post	Exercise versus Control Pre
Gender	6F, 2M		4F, 1M		
Duration of diabetes (months)	38.6 ± 6.9		17.2 ± 7.5		0.14
Age (years)	17.3 ± 0.8		15.3 ± 0.8		0.11
Anthropometric data					
Height (cm)	167.3 ± 4.3	167.7 ± 4.1	166.3 ± 3.8	166.8 ± 3.6	0.88
Weight (kg)	100.1 ± 9.6	100.1 ± 9.7	84.1 ± 9.4	86.5 ± 9.8**	0.29
Total fat (kg)	43.65 ± 6.59	42.74 ± 6.18	34.50 ± 4.79	36.46 ± 4.76*	0.34
Total lean (kg)	52.02 ± 3.40	53.36 ± 4.01**	45.55 ± 4.82	45.78 ± 4.94	0.28
% Fat	44.1 ± 2.8	43.2 ± 2.9	42.7 ± 2.4	44.0 ± 2.2	0.74
BMI kg m^−2^	36.1 ± 3.9	36.0 ± 3.8	30.0 ± 2.2	30.7 ± 2.4**	0.27
BMI *z*‐score	1.9 ± 0.2	1. 9 ± 0.2	1.8 ± 0.3	1.8 ± 0.3	0.72
Glycemic control					
HbA1c (%)	8.8 ± 1.0	9.2 ± 1.0	6.6 ± 0.2	6.5 ± 0.2	0.11
M (lbm; mg kg^−1^ min^−1^)†	4.7 ± 1.7	5.0 ± 1.5	5.7 ± 0.8	5.1 ± 1.2	0.66
Fitness and strength data					
$\dot{V}O_{2\mathrm{peak}}$ (mL kg^−1^ min^−1^)	25.7 ± 2.4	26.4 ± 3.5	29.9 ± 2.7	27.4 ± 2.7	0.28
Total strength (kg)	60.2 ± 7.4	91.6 ± 8.7**	60.2 ± 8.4	66.6 ± 9.8	0.99
Vascular data					
Brachial artery diameter (cm)	3.38 ± 0.18	3.49 ± 0.19	3.03 ± 0.21	2.97 ± 0.20	0.19
Max diameter post ischemia (cm)	3.65 ± 0.19	3.86 ± 0.25	3.27 ± 0.24	3.20 ± 0.20	0.244
FMD (%)	7.62 ± 1.2	9.82 ± 1.0*	7.84 ± 1.0	7.35 ± 1.1	0.91
Time to peak diameter (s)	68.4 ± 5.9	57.3 ± 11.7	57.7 ± 7.8	56.8 ± 11.2	0.29

*P < 0.05, **P < 0.01 compared to pre.

^†^ *n* = 6 in the exercise group and *n* = 4 in the control group for clamp data.

### Impact of exercise training on body weight and composition

**Figure 2:**
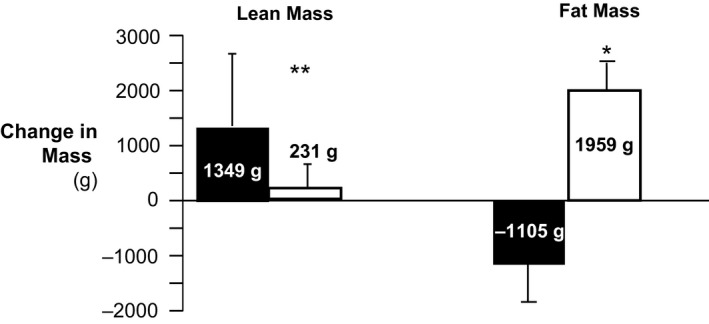
Body composition (change in total lean mass and total fat mass) in the exercise (black bars, *n* = 8) and control (white bars, *n* = 5) groups following the 12‐week intervention period.

There was no increase in body weight and BMI following the 12‐week intervention period in participants who received the exercise program, but a significant increase in weight and BMI was observed in the control group during this period. Body composition (DXA) measures revealed a significant effect on total fat mass (time × group interaction effect via two‐way ANOVA, *P* < 0.05), with a mean reduction of total fat mass by 1.10 kg in the exercise group and an increase of 1.96 kg in the control group. This was accompanied by a slight, although not statistically significant, change in fat‐free mass (+1.35 kg in the exercise group, compared to +0.23 kg increase in control group, Figure 2).

### Impact of exercise training on muscular strength and cardiorespiratory fitness

Combined upper and lower limb strength increased significantly as a result of the exercise training (time × group interaction effect via two‐way ANOVA, *P *<* *0.005). Post hoc analysis revealed that the combined strength increased by 31 kg in the exercise group (*P *<* *0.001) with no changes in combined strength in the control group (Table 1). Exercise training induced significant increases in lower limb strength (+23 kg, *P *<* *0.001) and pectoral strength (+14 kg, *P *<* *0.05) measures. No significant changes in any measures of muscular strength were observed in the control group following the 12‐week intervention period.

Cardiorespiratory fitness ($\dot{V}O_{2\mathrm{peak}}$) was not significantly altered following the intervention (time×group interaction effect via ANOVA *P* = 0.07). In agreement with this result, there was no significant change in HR_max_ (*P* = 0.886), maximal power (*P* = 0.980), or time to exhaustion (P = 0.812) following the intervention. Although statistical significance was not achieved, exercise training did attenuate the decrease in cardiorespiratory levels observed in the control group (−2.5 mL kg^−1^ min^−1^, Table 1).

### Impact of exercise training on vascular endothelial function

**Figure 3:**
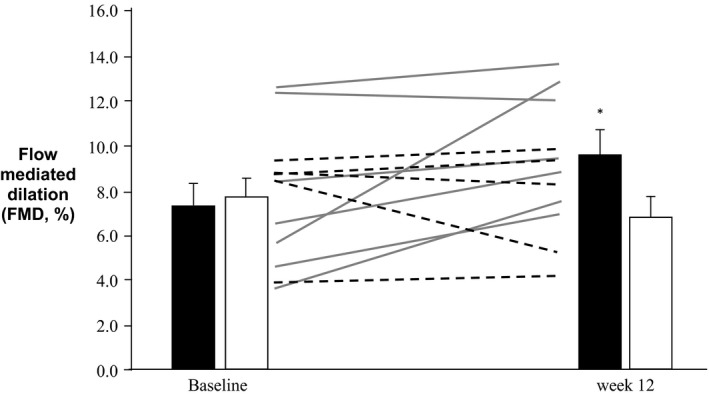
Mean conduit artery NO function (flow‐mediated dilation, FMD%) at baseline and week 12 in the exercise (black bars, *n* = 8) and control groups (white bars, *n* = 5). Individual data presented for the exercise group as gray lines, and dotted lines represents individual data for the control group. FMD% at week 12 was significantly increased compared to baseline in the exercise group (*P < 0.05).

**Figure 4:**
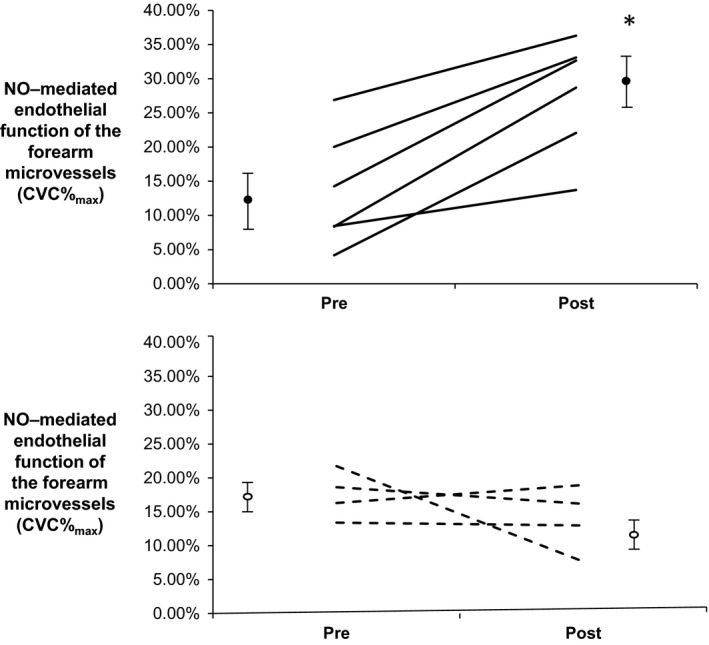
The change in contribution of NO‐mediated endothelial function in the microcirculation of the skin before and after exercise training in the exercise (top panel, *n* = 6) and control (bottom panel, *n* = 4) groups. Following week 12 of exercise training, there was a significant increase compared to baseline in the exercise group (*P < 0.05).

Following exercise training, ANOVA indicated that there was a significant time×group interaction effect in conduit artery endothelial function (FMD, *P* < 0.05). Post hoc analysis revealed a significant increase in FMD (+2.2 ± 1.1%, *P* < 0.05) following exercise training, while no changes were evident in the control group (Fig. 3). Brachial artery diameter did not change in either group following the intervention period (Table 1, *P* = 0.77, ANOVA). There was a significant time × group interaction effect in NO‐mediated microvascular function (CVC%_max_, ANOVA time × group interaction effect, *P* < 0.05), with a significant improvement following exercise training (*P* < 0.01, Fig. 4). The NO contribution to the response to localized heating was improved following the exercise intervention (Fig. 4, time × group interaction effect via two‐way ANOVA, *P *<* *0.05). No significant differences were observed in the control group (Fig. 4, *P* = 0.40, paired *t* test).

### Impact of exercise training on glycemic control

Whole body insulin sensitivity (M) did not significantly change in either group (Table 1), although the average decrease observed in the control group was attenuated by the exercise training intervention (Table 1).

### Impact of detraining

Following the completion of the intervention period, those in the exercise training group were followed for a further 12 weeks to assess persistence of the training. During this time, the exercise training sessions were stopped and participants were instructed to return to their normal activity levels. One individual from the exercise group was lost to follow‐up due to changes in medication (commencement of insulin). This participant did not receive the clamp or microvascular assessments at baseline and week 12.

Compared to values at entry into the study, no changes in either body weight or BMI were evident following the detraining period (Table 2). Total fat mass was stable following detraining, while the increase in fat‐free mass with training was largely maintained (Table 2). Increases in lower limb and upper body strength were maintained following training, whereas peak oxygen consumption ($\dot{V}O_{2\mathrm{peak}}$) did not change (Table 2).

**Table 2:** Impact of detraining (*n* = 7)

	Pretraining	Posttraining	Detraining
Anthropometric data			
Weight (kg)	97.7 ± 10.7	97.9 ± 11.5	97.6 ± 15.4
Total fat (kg)	42.17 ± 7.41	40.21 ± 7.08	40.43 ± 6.63
Total lean (kg)	51.07 ± 3.78	53.45 ± 4.9	53.35 ± 5.26
% Fat	43.6 ± 3.2	41. 9 ± 3.2	42.3 ± 2.8
BMI (kg m^−2^)	34.5 ± 4.1	34.5 ± 4.4	34.3 ± 4.3
BMI *z*‐score	1.8 ± 0.2	1.8 ± 0.2	1.8 ± 0.2
Glycemic control			
HbA1c (%)	8.5 ± 1.1	8.7 ± 1.7	8.8 ± 1.2
M (lbm; mg kg^−1^ min^−1^)†	4.7 ± 1.7	5.0 ± 1.5	4.0 ± 1.7
Fitness and strength data			
$\dot{V}O_{2\mathrm{peak}}$ (mL kg^−1^ min^−1^)	25.8 ± 2.9	27.8 ± 4.5	26.8 ± 3.4
Total strength (kg)	36.4 ± 5.4	60.0 ± 7.2*	57.9 ± 8.4**
Upper limb strength (kg)	26.3 ± 3.1	35.4 ± 4.3**	33.8 ± 4.2**
Lower limb strength (kg)	62.7 ± 8.1	95.4 ± 9.7*	91.7 ± 11.2**
Vascular data			
Brachial artery diameter (cm)	3.49 ± 0.14	3.60 ± 0.21	3.34 ± 0.21
Max diameter post ischemia (cm)	3.77 ± 1.68	3.99 ± 2.40	3.61 ± 2.47
FMD (%)	8.07 ± 1.25	10.26 ± 0.97	8.21 ± 1.14
Time to peak diameter (s)	70.1 ± 6.40	57.4 ± 13.5	40.33 ± 15.24
Microvessel endothelial function (%CVC_max_)†	13.7 ± 3.5	27.8 ± 3.4**	11.4 ± 0.8

*P < 0.05, **P < 0.01 compared to baseline data.

^†^ *n* = 6 for these measures.

The improvement in conduit artery FMD following exercise training were not sustained following detraining. Similarly, improvements noted following the training program in stimulated microvascular endothelial function returned to baseline levels following the detraining period (11.4 ± 0.8 CVC%_max_).

No significant changes were observed in relation to whole body insulin sensitivity (M) following the detraining period (Table 2, one‐way ANOVA, *P* = 0.6).

## Discussion

In our cohort of adolescents with type 2 diabetes, a 12‐week structured exercise training program had beneficial impacts on measures of conduit and microvessel endothelial function, body composition, and strength. Our findings complement previous reports that exercise training can improve vascular function in adults with type 2 diabetes (Maiorana et al. 2001) and in obese, nondiabetic children (Watts et al. 2004b) and adolescents (Watts et al. 2004a). However, this is the first study to report that arterial function can be improved as a result of exercise training in adolescents with type 2 diabetes. This is particularly important in this clinical population, who are at highly elevated risk of developing vascular disease (Eppens et al., 2006; Naylor et al. 2011).

Previously, Maiorana et al. (2001) demonstrated improved endothelial function in both resistance and conduit vessels following 8 weeks of supervised exercise training in 16 adults with type 2 diabetes. Endothelial function of the resistance vessels was assessed using forearm plethysmography with concomitant intrabrachial infusions of acetylcholine, ACh, while FMD was employed to assess conduit artery endothelial function. Of note, smooth muscle mediated vasodilation (analyzed via the use of SNP and GTN for resistance and conduit arteries, respectively) were unchanged following the training program. Cohen et al. (2008) also reported that a 14‐month progressive resistance training program improved microvessel endothelial function in adults with type 2 diabetes. In this study, endothelial function was assessed in the skin microcirculation using laser Doppler flow following iontophoresis of acetylcholine (ACh) and sodium nitroprusside (SNP). To the contrary, Middlebrook et al. (2006) reported that 6 months of aerobic training did not improve endothelial function in adult skin microvessels (age 62.9 ± 7.6 years) of type 2 diabetics with good glycemic control (HbA1c 6.8 ± 0.9%). In that study, microvessel function was assessed using the maximum skin hyperemia to local heating and endothelial and nonendothelial responsiveness following the iontophoretic application of acetylcholine (ACh) and sodium nitroprusside (SNP). It is also worthwhile noting that, similar to our findings, no improvements were seen in $\dot{V}O_{2\max}$ following the exercise intervention. Exercise training studies have also shown improvement in plasma biomarkers of inflammation and endothelial function in older adults with type 2 diabetes (Zoppini et al. 2006). In this cohort, the authors showed improvements unrelated to changes in body weight, waist circumference, blood pressure, HbA1c, plasma triglyceride, or LDL cholesterol concentrations, in keeping with our findings. The discrepancies noted above may, at least in part, be attributed to the variance in training programs and patient populations, however, overall they provide support of our current findings in an adolescent cohort.

In addition, the present study reports a significant improvement in the NO contribution to microvascular function. This may have some clinical relevance, given evidence indicating that cutaneous microvessel dysfunction is correlated with coronary endothelial dysfunction (Shamim‐Uzzaman et al. 2002; Khan et al. 2008) and cardiovascular risk factors (Carberry et al. 1992; Khan et al. 1999; Rizzoni et al. 2003). The improvements in NO‐mediated function observed in this study therefore support the use of exercise training to prevent future microvascular disease in this population of young individuals.

Pediatric populations are distinctly different in terms of physiology from their adult counterparts. For example, puberty induces increased resistance to the action of insulin, resulting in relative hyperinsulinemia (Moran et al. 1999), and after puberty, basal and stimulated insulin responses decline. In the presence of normal pancreatic cell function, puberty‐related insulin resistance is compensated by increased insulin secretion. It is therefore important to emphasize that these findings are the first in young people with type 2 diabetes to demonstrate that vascular function measures, collected by distinct techniques and at different levels of the arterial tree, consistently increased following exercise training.

In our study, exercise training also produced modest benefits in terms of body composition. Notably, these improvements were not reflected in terms of changes in either total body weight or BMI. This is similar to findings reported by Watts et al. (2004a), where there were no changes in either total weight or BMI as a result of exercise training, but significant improvements in adiposity and muscle mass observed following an 8‐week exercise training intervention in 19 obese adolescents (14.3 ± 1.5 years, mean BMI 34.4 ± 0.8). Collectively, these findings emphasize the importance of comprehensive assessment of body composition, rather than reliance on body weight or BMI as primary outcome measures in exercise intervention studies.

We did not observe significant changes in insulin sensitivity in this study. It is well established that exercise training is an effective strategy to *prevent* diabetes (Diabetes Prevention Program Group 2002) and we have previously reported that a similar training protocol induces improvements in insulin sensitivity in obese, nondiabetic adolescents (Bell et al. 2007). However, there is very little published data regarding the impact of physical activity and exercise training in adolescents with established type 2 diabetes. In accordance with our finding, the Today Study Group (2012) reported that in 10‐ to 17‐year‐olds with type 2 diabetes, the addition of a lifestyle intervention to medication did not produce superior benefits compared to treatment by medication alone. Failure to achieve durable glycemic control (HBA1c >8%) was reported in 46.6% of those in the metformin plus lifestyle intervention, compared to 51.7% of those who only received metformin. The sole use of a lifestyle intervention was unsuccessful in controlling glucose metabolism in German and Austrian children and adolescents with established type 2 diabetes (Reinehr et al. 2008). These studies differ importantly from the findings of the diabetes prevention study, in which adults showed nearly twice the benefit of metformin as a result of lifestyle modification involving exercise (Diabetes Prevention Program Research Group, 2002). Aside from revealing a possible distinction between studies of children and adults with diabetes, these studies also emphasize the importance of promoting a healthy lifestyle and regular exercise in obese young people before they develop type 2 diabetes, when improvements in glycemic control may be more difficult to achieve via exercise training. Notwithstanding the absence of change in insulin sensitivity, it is important to reiterate that beneficial adaptations were apparent in both conduit and microvascular function as a consequence of exercise training in our study.

While improvements in insulin sensitivity were not significant in the exercise group, there was a nonsignificant decrease in the control group, accompanied by a significant increase in fat mass. This implies that, while exercise training did not reveal significant improvements in insulin sensitivity in our modest study sample, it may attenuate the natural progression of type 2 diabetes and its complications in this adolescent cohort. There is no doubt the small sample size in this mechanistic experiment limits our ability to draw definitive conclusions regarding the impact of diabetes and exercise training on glycemic control, but the trends that have emerged should encourage larger funded trials of these outcomes.

It is also interesting to note that, while we observed significant strength gains in our cohort, no changes were seen in terms of cardiorespiratory fitness following the exercise intervention. While this may seem unexpected, we are not the first group to report this. Recent data even suggest that individuals with a first degree relative with type 2 diabetes have a lower response in terms of fitness gain, compared to those without a family history (Ekman et al. 2015).

Finally, we also assessed detraining data. These data indicate that the favorable changes in body composition and strength induced by the exercise training can be maintained beyond the duration of the training program. However, vascular function declined following the cessation of training, suggesting that a continuation of the stimulus to vascular adaptation, likely to be shear stress (Joyner and Green 2009; Tinken et al. 2009), may be necessary to maintain gains in arterial health.

To monitor compliance, all exercise training sessions were supervised by clinical exercise physiologists and personalized programs were designed for each participant. Another strength of the study is the inclusion of a control group of subjects who undertook routine clinical care, which allowed us to determine the natural progression of type 2 diabetes in adolescents. Limitations of this study include our small sample size. Although the study was adequately powered to detect the magnitude of observed changes in the primary outcome measures (conduit and microvessel function) (Woodman et al. 2001), the comparison between the exercise intervention group and control group should be interpreted with caution due to differences between the groups at baseline. It is worthwhile noting that the participants in our study possessed poor metabolic control (average HbA1c of all participants at entry = 8%), and that although the difference in HbA1c at entry was not statistically significant, differences were noted between the groups. Nonetheless, the control group deteriorated across the intervention period, while the trained group, who exhibited worse physiological function and health at baseline, demonstrated exercise training mediated improvements. For example, despite the control group being leaner and having a lower body weight at baseline, there were significant increases in weight, which occurred alongside a reduction in insulin sensitivity (M), indicating a decline in health over the 12‐week intervention period.

In conclusion, we present data in a sample of subjects suggesting that exercise training interventions can improve both conduit and microvascular endothelial function and health in adolescents with type 2 diabetes. Detraining data imply that continuous training is likely to be necessary to maintain these clinically important benefits. We conclude that personalized and intensive exercise training may be effective in ameliorating the detrimental clinical impacts of type 2 diabetes and may decrease the future risk of cardiovascular complications in this very high‐risk cohort.

## Conflict of Interest

None declared.

## References

[phy212713-bib-0001] Alberti, G. , P. Zimmet , J. Shaw , Z. Bloomgarden , F. Kaufman , and M. Silink ; for the International Diabetes Federation Consensus Workshop . 2004 Type 2 diabetes in the young: the evolving epidemic. Diabetes Care 27:1798–1811. 15220270 10.2337/diacare.27.7.1798

[phy212713-bib-0002] American Diabetes Association . 2011 Standards of medical care in diabetes – 2011. Diabetes Care 34:S11–S61. 21193625 10.2337/dc11-S011 PMC3006050

[phy212713-bib-0003] Bell, L. , K. Watts , A. Siafarikas , A. Thompson , N. Ratnam , M. Bulsara , et al. 2007 Exercise alone reduces insulin resistance in obese children independently of changes in body composition. J. Clin. Endocrinol. Metab. 92:4230–4235. 17698905 10.1210/jc.2007-0779

[phy212713-bib-0004] Black, M. A. , D. J. Green , and N. T. Cable . 2008 Exercise prevents age‐related decline in nitric‐oxide‐mediated vasodilator function in cutaneous microvessels. J. Physiol. 586:3511–3524. 18483071 10.1113/jphysiol.2008.153742 PMC2538814

[phy212713-bib-0005] Carberry, P. A. , A. M. Shepherd , and J. M. Johnson . 1992 Resting and maximal forearm skin blood flows are reduced in hypertension. Hypertension 20:349–355. 1516954 10.1161/01.hyp.20.3.349

[phy212713-bib-0006] Cohen, N. D. , D. Dunstan , C. Robinson , E. Vulikh , P. Zimmet , and J. Shaw . 2008 Improved endothelial function following a 14‐month resistance exercise training program in adults with type 2 diabetes. Diabetes Res. Clin. Pract. 79:405–411. 18006170 10.1016/j.diabres.2007.09.020

[phy212713-bib-0500] Colberg, S. R. , R. J. Sigal , B. Fernhall , J. G. Regensteiner , B. J. Blissmer , R. R. Rubin , et al. 2010 Exercise and type 2 diabetes. The American College of Sports Medicine and the American Diabetes Association: joint position statement. Diabetes Care. 33:e147–e167. 21115758 10.2337/dc10-9990 PMC2992225

[phy212713-bib-0007] Colberg, S. , K. Stansberry , P. McNitt , and A. Vinik . 2002 Chronic exericse is associated with enhanced cutaneous blood flow in Type 2 diabetes. J. Diabetes Complications 16:139–145. 12039396 10.1016/s1056-8727(01)00222-7

[phy212713-bib-0009] Copeland, K. , J. Silverstein , K. Moore , G. Prazar , T. Raymer , R. Shiffman , et al. 2013 Clinical practice guideline. Management of newly diagnosed type 2 diabetes mellitus (T2DM) in children and adolescents. Pediatrics 131:364–382. 23359574 10.1542/peds.2012-3494

[phy212713-bib-0010] Cracowski, J. L. , C. T. Minson , M. M. Salvat , and J. R. Halliwill . 2006 Methodological issues in the assessment of skin microvascular endothelial function in humans. Trends Pharmacol. Sci. 27:503–508. 16876881 10.1016/j.tips.2006.07.008

[phy212713-bib-0011] Davis, E. A. , M. Russell , and T. W. Jones . 2002 Increasing prevalence of type 2 diabetes in Australian children. Aust Diabetes Soc Annual Conference, Adelaide, September 2002.

[phy212713-bib-0012] DeFronzo, R. A. , J. D. Tobin , and R. Andres . 1979 Glucose clamp technique: a method for quantifying insulin secretion and resistance. Am. J. Physiol. 237:E214–E223. 382871 10.1152/ajpendo.1979.237.3.E214

[phy212713-bib-0013] Diabetes Prevention Program Research Group . 2002 Reduction in the incidence of type 2 diabetes with lifestyle intervention or metformin. N. Engl. J. Med. 346:393–403. 11832527 10.1056/NEJMoa012512 PMC1370926

[phy212713-bib-0014] Ekman, C. , T. Elgzyri , K. Ström , P. Almgren , H. Parikh , M. Dekker Nitert , et al. 2015 Less pronounced response to exercise in healthy relatives to type 2 diabetics compared to controls. J. Appl. Physiol. 119:953–960. 26338460 10.1152/japplphysiol.01067.2014 PMC4816086

[phy212713-bib-0015] Eppens, C. M. , E. M. Craig , J. Cusumano , S. Hing , K. A. Chan , J. N. Howard , et al. 2006 Prevalence of diabetes complications in adolescents with type 2 compared with type 1 diabetes. Diabetes Care 29:1300–1306. 16732012 10.2337/dc05-2470

[phy212713-bib-0016] Ferguson, M. A. , B. Gutin , N. Le , W. Karp , M. Litaker , M. Humphries , et al. 1999 Effects of exercise training and its cessation on components of the insulin resistance syndrome in obese children. Int. J. Obes. Relat. Metab. Disord. 22:889–895. 10490792 10.1038/sj.ijo.0800968

[phy212713-bib-0017] Hotu, S. , B. Carter , P. D. Watson , W. S. Cutfield , and T. Cundy . 2004 Increasing prevalence of type 2 diabetes in adolescents. J. Paediatr. Child Health 40:201–204. 15009549 10.1111/j.1440-1754.2004.00337.x

[phy212713-bib-0018] Jones, H. , N. C. Lewis , A. Thompson , K. Marrin , D. J. Green , and G. Atkinson . 2012 Diurnal variation in vascular function: role of sleep. Chronobiol. Int. 29:271–277. 22390240 10.3109/07420528.2012.654554

[phy212713-bib-0019] Joyner, M. J. , and D. J. Green . 2009 Exercise protects the cardiovascular system: effects beyond traditional risk factors. J. Physiol. 1:5551–5558. 19736305 10.1113/jphysiol.2009.179432 PMC2805367

[phy212713-bib-0020] Kaufman, F. R. 2002 Type 2 diabetes in children and young adults: a “new epidemic”. Clinical Diabetes 20:217–218.

[phy212713-bib-0021] Khan, F. , S. J. Litchfield , P. A. Stonebridge , and J. J. Belch . 1999 Lipid‐lowering and skin vascular responses in patients with hypercholesterolaemia and peripheral arterial obstructive disease. Vasc. Med. 4:233–238. 10613627 10.1177/1358836X9900400405

[phy212713-bib-0022] Khan, F. , D. Patterson , J. J. Belch , K. Hirata , and C. C. Lang . 2008 Relationship between peripheral and coronary function using laser Doppler imaging and transthoracic echocardiography. Clin. Sci. (Lond.) 115:295–300. 18338981 10.1042/CS20070431

[phy212713-bib-0023] Krakoff, J. , R. Lindsay , H. Looker , R. G. Nelson , R. L. Hanson , and W. C. Knowler . 2003 Incidence of retinopathy and nephropathy in youth‐onset compared with adult‐onset type 2 diabetes. Diabetes Care 26:76–81. 12502661 10.2337/diacare.26.1.76

[phy212713-bib-0024] Maiorana, A. , G. O'Driscoll , C. Cheetham , L. Dembo , K. Stanton , C. Goodman , et al. 2001 The effect of combined aerobic and resistance exercise training on vascular function in type 2 diabetes. J. Am. Coll. Cardiol. 38:860–866. 11527646 10.1016/s0735-1097(01)01439-5

[phy212713-bib-0025] Middlebrook, A. , L. Elston , K. Macleod , D. Mawson , C. Ball , A. Shore , et al. 2006 Six months of aerobic exercise does not improve microvascular function in type 2 diabetes mellitus. Diabetologia 49:2263–2271. 16944096 10.1007/s00125-006-0361-x

[phy212713-bib-0026] Moran, A. , D. Jacobs , J. Steinberger , C. Hong , R. Prineas , R. Luepker , et al. 1999 Insulin resistance during puberty. Results from clamp studies in 357 children. Diabetes 48:2039–2044. 10512371 10.2337/diabetes.48.10.2039

[phy212713-bib-0027] Naylor, L. H. , D. J. Green , T. W. Jones , R. J. Kalic , K. L. Suriano , M. Shah , et al. 2011 Endothelial function and carotid intima‐medial thickness in adolescents with type 2 diabetes mellitus. J. Pediatr. 159:971–974. 21722916 10.1016/j.jpeds.2011.05.019

[phy212713-bib-0028] Reinehr, T. , E. Schober , C. L. Roth , S. Wiegand , and R. Holl . 2008 Type 2 diabetes in children and adolescents in a 2‐year follow‐up: insufficient adherence to diabetes centers. Horm. Res. Paediatr. 69:107–113. 10.1159/000111814 18059091

[phy212713-bib-0029] Riddoch, C. 1998 Relationships between physical activity and physical health in young people Pp. 17–48 in Biddle S., Saddle J., Cavill N., eds. Young and active? Young people and health enhancing physical activity ‐ evidence and implications. Health Education Authority, London.

[phy212713-bib-0030] Rizzoni, D. , E. Porteri , G. E. Boari , C. De Ciuceis , I. Sleiman , M. L. Muiesan , et al. 2003 Prognostic significance of small‐artery structure in hypertension. Circulation 108:2230–2235. 14557363 10.1161/01.CIR.0000095031.51492.C5

[phy212713-bib-0031] Ross, R. 1993 The pathogenesis of atherosclerosis: a perspective for the 1990s. Nature 362:801–809. 8479518 10.1038/362801a0

[phy212713-bib-0032] Shamim‐Uzzaman, Q. A. , D. Pfenninger , C. Kehrer , A. Chakrabarti , N. Kacirotti , M. Rubenfire , et al. 2002 “Altered cutaneous microvascular responses to reactive hyperaemia in coronary artery disease: a comparative study with conduit vessel responses. Clin. Sci. (Lond.) 103:267–273. 12193152 10.1042/cs1030267

[phy212713-bib-0033] Sinha, R. , G. Fisch , B. Teague , W. V. Tamborlane , B. A. Banyas , M. Savoye , et al. 2002 Prevalence of impaired glucose tolerance among children and adolescents with marked obesity. N. Engl. J. Med. 346:802–810. 11893791 10.1056/NEJMoa012578

[phy212713-bib-0034] Thijssen, D. H. , M. A. Black , K. E. Pyke , J. Padilla , G. Atkinson , R. A. Harris , et al. 2011 Assessment of flow‐mediated dilation in humans: a methodological and physiological guideline. Am. J. Physiol. Heart Circ. Physiol. 300:H2–H12. 20952670 10.1152/ajpheart.00471.2010 PMC3023245

[phy212713-bib-0035] Tinken, T. M. , D. H. Thijssen , N. Hopkins , M. A. Black , E. A. Dawson , C. T. Minson , et al. 2009 Impact of shear rate modulation on vascular function in humans. Hypertension 54:278–285. 19546374 10.1161/HYPERTENSIONAHA.109.134361 PMC3012006

[phy212713-bib-0036] Today Study Group . 2012 A clinical trial to maintain glycemic control in youth with type 2 diabetes. N. Engl. J. Med. 366:2247–2256. 22540912 10.1056/NEJMoa1109333 PMC3478667

[phy212713-bib-0038] Watts, K. , P. Beye , A. Siafarikas , E. A. Davis , T. W. Jones , G. O'Driscoll , et al. 2004a Exercise training normalizes vascular dysfunction and improves central adiposity in obese adolescents. J. Am. Coll. Cardiol. 43:1823–1827. 15145107 10.1016/j.jacc.2004.01.032

[phy212713-bib-0039] Watts, K. , P. Beye , A. Siafarikas , G. O'Driscoll , T. W. Jones , E. A. Davis , et al. 2004b Effects of exercise training on vascular function in obese children. J. Pediatr. 144:620–625. 15126996 10.1016/j.jpeds.2004.02.027

[phy212713-bib-0040] Woodman, R. J. , D. A. Playford , G. F. Watts , C. Cheetham , C. Reed , R. R. Taylor , et al. 2001 Improved analysis of brachial artery ultrasound using a novel edge‐detection software system. J. Appl. Physiol. 91:929–937. 11457812 10.1152/jappl.2001.91.2.929

[phy212713-bib-0041] Yokoyama, H. , M. Okudaira , T. Otani , A. Sato , J. Miura , H. Takaike , et al. 2000 Higher incidence of diabetic nephropathy in type 2 than type 1 diabetes in early‐onset diabetes in Japan. Kidney Int. 58:302–311. 10886575 10.1046/j.1523-1755.2000.00166.x

[phy212713-bib-0042] Zeitler, P. , J. Fu , N. Tandon , K. Nadeau , T. Urakami , T. Barlett , et al. 2014 Type 2 diabetes in the child and adolescent. ISPAD clinical practice consensus guidelines 2014 compendium. Pediatr. Diabetes 15:26–46. 25182306 10.1111/pedi.12179

[phy212713-bib-0043] Zoppini, G. , G. Targher , C. Zamboni , C. Venturi , V. Cacciatori , P. Moghetti , et al. 2006 Effects of moderate‐intensity exercise training on plasma biomarkers of inflammation and endothelial dysfunction in older patients with type 2 diabetes. Nutr. Metab. Cardiovasc. Dis. 16:543–549. 17126770 10.1016/j.numecd.2005.09.004

